# Quantifying Discretization Errors in Electrophoretically-Guided Micro Additive Manufacturing

**DOI:** 10.3390/mi9090447

**Published:** 2018-09-07

**Authors:** David Pritchet, Newell Moser, Kornel Ehmann, Jian Cao, Jiaxing Huang

**Affiliations:** 1 Mechanical Engineering Department, Northwestern University, Evanston, IL 60208, USA; newellmoser2018@u.northwestern.edu (N.M.); k-ehmann@northwestern.edu (K.E.); jcao@northwestern.edu (J.C.); 2 Materials Science and Engineering Department, Northwestern University, Evanston, IL 60208, USA; jiaxing-huang@northwestern.edu

**Keywords:** control design, design methodology, dielectrophoresis, electrophoretic deposition, error analysis, finite element analysis

## Abstract

This paper presents process models for a new micro additive manufacturing process termed Electrophoretically-guided Micro Additive Manufacturing (EPμAM). In EPμAM, a planar microelectrode array generates the electric potential distributions which cause colloidal particles to agglomerate and deposit in desired regions. The discrete microelectrode array nature and the used pulse width modulation (PWM) technique for microelectrode actuation create unavoidable process errors—space and time discretization errors—that distort particle trajectories. To combat this, we developed finite element method (FEM) models to study trajectory deviations due to these errors. Mean square displacement (MSD) analysis of the computed particle trajectories is used to compare these deviations for several electrode geometries. The two top-performing electrode geometries evaluated by MSD were additionally investigated through separate case studies via geometry variation and MSD recomputation. Furthermore, separate time-discretization error simulations are also studied where electrode actuating waveforms were simulated. The mechanical impulse of the electromechanical force, generated from these waveforms is used as the basis for comparison. The obtained results show a moderate MSDs variability and significant differences in the computed mechanical impulses for the actuating waveforms. The observed limitations of the developed process model and of the error comparison technique are briefly discussed and future steps are recommended.

## 1. Introduction

Electric field guided particle manipulation and deposition is a promising and versatile micro- and nano-fabrication technique [1,2]. The versatility of particle manipulation with electric fields stems from a multitude of fitting electrode array geometries and actuation strategies [3], and from the application of suitable electric signals to produce particle deposits with desirable characteristics [4]. During the design and implementation phases of a desired particle deposition system, it is important to account for any process errors that arise from the selection of electrode geometry and errors that originate in the control system of such devices so that the desired accuracy and precision of the system can be achieved. On a practical note, during the deposition or manipulation process, these errors result in the particles’ deviation from their intended paths that can usually be tracked with microparticle image velocimetry (µPIV) techniques [5,6,7,8]. But µPIV has limitations which are related to particle size and concentration, the diffraction limit of the sensors [9,10,11], or to the implementation of the tracking algorithm [12,13]. A promising, design-based alternative for the characterization of the mentioned errors and their influence on particle trajectories is multiphysics finite element analysis (FEA) which is commonly used in the design stages of microelectromechanical systems (MEMS) [14,15,16] or for the optimization of (micro)antenna geometries [17,18].

In this paper, we present multi-physics FEA to characterize and quantify the arising process errors in the case of a novel micro-additive manufacturing process [19,20] currently under development and which will be explained in the next section. The employed finite element method (FEM) is used to compute the electric field distribution for given microelectrode geometries and to simulate a single deposition cycle of the new micro-additive manufacturing process. This deposition cycle would be continuously repeated until the desired particle distribution deposit is obtained, similar to the colloidal crystal layering technique [21]. The particle trajectories obtained in the single deposition cycle are analyzed with the mean square displacement (MSD) technique, commonly used in the analysis of colloidal solutions [22,23,24,25,26,27,28]. From the MSD results, the top performing microelectrode geometry candidates are selected for closer analysis. The effects of different actuating waveforms are addressed through a separate numerical analysis.

In the remainder of this introduction section, the novel micro additive manufacturing process with electric fields is presented, followed by a subsection on the implemented control algorithm—where two types of discretization errors are defined—and concluded with descriptions of the realized numerical studies. The methods section explains in detail all the steps used in building the FEA studies for the two types of discretization errors. Next, the results and discussion sections present and describe the obtained results for the two study types and additionally report two case studies of parametric sweeps for two electrode geometries selected via a comparative design matrix. Finally, the conclusion section summarizes the obtained results, comments on the study’s limitations, and suggests the viable next steps.

### 1.1. Electrophoretically-Guided Micro Additive Manufacturing (EPµAM) Process

**Figure 1:**
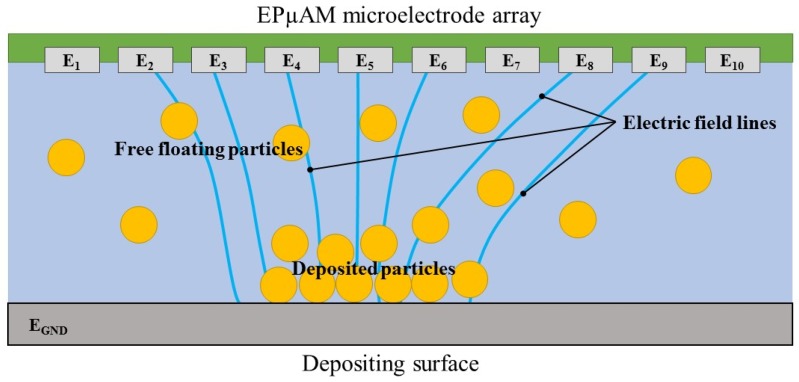
Electrophoretically-Guided Micro Additive Manufacturing (EPµAM) Process illustration.

The new EPμAM process uses an array of individually addressable microelectrodes to manipulate and deposit particles suspended in a colloidal solution. Individual microelectrode actuation allows the generation of the desired electric field’s distribution. These fields, due to dielectrophoretic phenomena [29,30,31,32,33,34], control particle positions and electrophoretically [35,36,37,38] deposit them. The process schematic—as a cross-section of the EPμAM apparatus—is shown in Figure 1.

The operating principle of this device is based on the application of a suitable electric potential to each individual electrode (E_1_–E_10_) located on the top of the figure. These microelectrodes are embedded on the surface of a MEMS device, which for clarity purposes, was omitted from the figure. However, the fabrication limitations of the said MEMS devices play a decisive role in the EPµAM process error estimation. After the application of the electric potential to the microelectrodes, a non-uniform electric field, both in time and space, is created between the microelectrode array and the ground electrode (E_GND_). When particles are introduced into this field via colloidal solutions, they start to migrate towards the electric field extrema. Based on the material properties of the particles and of the surrounding fluid, they may migrate towards the electric field maxima (i.e., microelectrode surfaces) or electric field minima (i.e., away from the microelectrode surfaces). This maxima/minima behavior can be tuned and controlled with the frequency of the applied electric fields and is explained by the dielectrophoresis phenomenon [39,40,41].

The grouping, migration, and diffusion of particle groups in the EPμAM process can be described with the Nernst-Planck equation [42]:

(1) $$\partial c/\partial t=-\nabla \cdot \left(-D\nabla c+\vec{u}c\right)$$

where *c* is the concentration of the particles/species, *t* is time, ∇ is the gradient operator, *D* is the diffusion coefficient and $\vec{u}$ is the total velocity of the species, defined as [42]:

(2) $$\vec{u}={\vec{u}}_{fluid}+{\vec{u}}_{EP}$$

The first right hand side (RHS) term represents the velocity of the fluid while the second term is the electromigratory velocity of the species/particles which is the product of the electrophoretic mobility $\mu_{EP}$, and of the electric field $\vec{E}$ [42]:

(3) $${\vec{u}}_{EP}=\mu_{EP}\vec{E}$$

In the case of small work volumes and in regions sufficiently far away from the electrodes, the fluid velocity can be neglected, leaving only the electromigratory term in (2). If necessary, the exact fluid flow solution can be derived from Brady’s investigation [43] to account for this term. Considering the scope of this paper, this term is neglected to simplify the predominantly electrodynamic analysis of particle actuation via electric fields.

When the Nernst-Planck equation is applied to a control volume (e.g., the workspace of the EPμAM apparatus) it prescribes the conservation of the species. If the Nernst-Planck equation (1) is weighted by the product of the species valence and Faraday’s constant then the same equation leads to the charge conservation equation [42]:

(4) $$\partial \rho_{E}/\partial t=-\nabla \cdot \left(-D\nabla \rho_{E}+\sigma \vec{E}\right)$$

In the context of the EPμAM process, (4) describes the evolution of the net electric charge density, $\rho_{E}$, driven by diffusion (first term on the RHS) and convection (second term on the RHS) due to the applied electric field. The coefficient *σ* is the molar conductivity of the species/particles. Because the EPμAM process uses a colloidal suspension of particles, the net electric charge density is directly related to the local particle concentration. From the process control perspective, the EPμAM process uses electric fields to combat particle diffusion processes and ultimately to rearrange the particles in the desired configuration.

### 1.2. EPµAM Control Algorithm

The free-floating particles in Figure 1 represent the initial positions—the initial state of the system. The particles deposited on the bottom electrode portray the final, desired state of the system. The goal of the developed control algorithm for the EPμAM process [20] is to compute the appropriate electric potential distribution based on the initial and the desired final positions of the particles. The computed electric potential is the solution of the inverse kinematic problem (IKP) of the EPμAM device, which is the main output of the control algorithm described in this subsection. During the particle deposition process in EPμAM, this electric potential is computed by the device controller and then continuously applied to the microelectrode array (electrodes E_1_–E_10_ in Figure 1). This implemented control algorithm is briefly explained here.

The initial step of the control algorithm is to compute the representative distributions (i.e., their probability density functions) of the initial and final particle groups. For the sake of simplicity, a normal (Gaussian) one-dimensional distribution is used here:

(5) $$n\left(x\right)=\left(1/\left(\sqrt{2\pi S^{2}}\right)\right)exp\left(-{\left(x-\mu \right)}^{2}/\left(2S^{2}\right)\right)$$

where *S* is the standard deviation and *µ* is the mean of the distribution. Other distribution types are also valid. After computation of the initial and final distributions, the Boltzmann’s law equation [44] is used to compute the required distribution of the potential energy:

(6) $$n=n_{0}exp\left(E_{POT}/k_{b}T\right)$$

where $n_{0}$ and *n* are the initial and final distributions, respectively, *k_b_* is Boltzmann’s constant and *T* is the system temperature, assumed to be constant, and *E_POT_* is the sought for ideal potential energy distribution. The previous equation is reordered to express the ideal potential energy distribution and is normalized with the particle’s viscous mobility coefficient $\mu_{visc}$ to account for particle drag in the fluid, i.e.,

(7) $$E_{POT}=\mu_{visc}k_{b}T\;ln\left(n/n_{0}\right)$$

The final step in this control algorithm is to convert the obtained potential energy distribution into values of the electric potential that need to be applied to the microelectrodes. This is accomplished by equating the computed ideal potential energy distribution with the energy for the planar capacitor *E_CAP_*:

(8) $$E_{CAP}=CV^{2}/2$$

where *V* is the electric potential and *C* is the capacitance of the system, given by [45]:

(9) $$C=\varepsilon_{0}\varepsilon_{r}A/d$$

In (9), $\varepsilon_{0}$ and $\varepsilon_{r}$ are electric permittivities of vacuum and dielectric fluid, respectively, *A* is the area of the microelectrode array, and *d* is the distance between the microelectrode array and the ground electrode. The desired electric potential is obtained by first equating (7) and (8) and then solving the obtained equation for the electric potential distribution *V_ideal_* to yield:

(10) $$V_{ideal}=\sqrt{2\mu_{visc}\left(k_{b}T/C\right)ln\left(n/n_{0}\right)}$$

**Figure 2:**
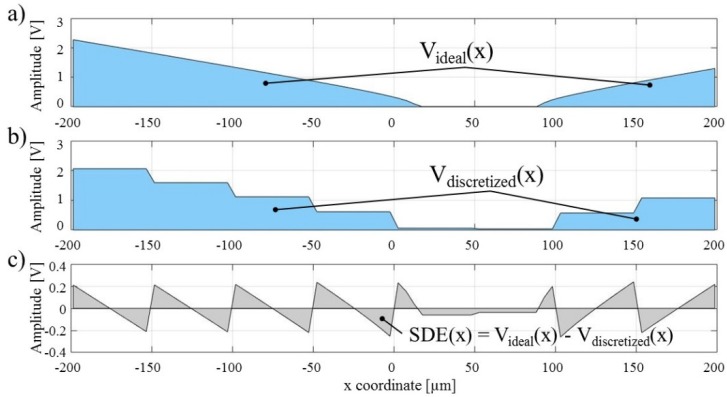
Example electric potential distributions (**a**) Ideal case; (**b**) Discretized case; (**c**) Space discretization error (SDE).

Figure 2a shows an example of a computed electric potential distribution (i.e., the real part of the solution). At this point, it is worth noting that this distribution value is for the ideal case. In the real, physical world the microelectrodes responsible for generating this distribution have a finite size and have finite distances between themselves. Moreover, the conductive material from which the microelectrodes are made cause the electric charge to be immediately redistributed across the electrode surface [45], which, in turn, causes the electric potential distribution of the discretized case to look like it does in Figure 2b (e.g., flat, stepwise shape pattern, as opposed to the smooth potential depicted in Figure 2a). This is an unfortunate and unavoidable result of the discretized nature of microelectrode arrays. The difference between the ideal (Figure 2a) and discretized (Figure 2b) distribution of the electric potential is shown in Figure 2c and is here defined as the space discretization error (SDE). It is important to quantify and minimize this error so that the desired accuracy and efficiency of the EPμAM process can be achieved.

**Figure 3:**
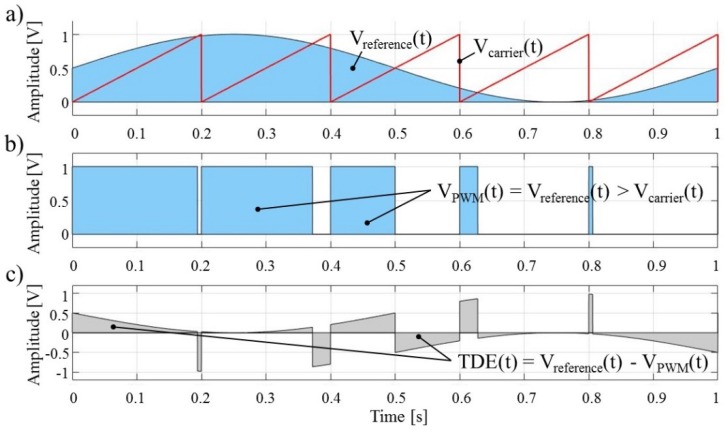
(**a**) Reference and carrier signal waveforms; (**b**) Pulse width modulation (PWM)-obtained pulse train; (**c**) Time discretization error (TDE).

In the general case, the timely application of different electric potentials to each microelectrode in the microelectrode array during the deposition process require some form of power modulation technique with a high degree of scalability and reliability—the commonly used technique is pulse width modulation (PWM) [46]. In PWM, the reference signal, in this case, the desired waveform to be applied to the microelectrode, is modulated with a carrier signal of a higher frequency—commonly used is the sawtooth waveform (Figure 3a). The output from this modulation technique is a pulse train with two values (high and low value) which can readily be implemented as a digital signal output on the majority of (micro)controller platforms used in process machines (Figure 3b). Although the power of the reference signal and of the resulting pulse train waveform is essentially the same, the difference in shapes of these waveforms may induce different forces in cases of small particles and for high frequencies, such as the ones used in electrophoretic deposition or in the EPμAM process. Because of this, we are defining the difference between the reference signal waveform and the pulse train waveform as the time discretization error (TDE) (Figure 3c). Understanding the size of this error and of the effects it has on particle mobility will decrease the chance of oversupplying the EPμAM system with an electric potential, which will directly improve the durability of the EPμAM device and increase the overall efficiency of the process.

The objective of this paper is to numerically investigate and quantify two types of errors that stem from the use of modulated electric fields. The first type of error is caused by the finite size of the electrodes in the microelectrode array; it is unavoidable and it can only be minimized by selection of the proper electrode geometry. This selection process is presented in the Space Discretization Error studies and their subsequent analysis. The second type of discretization error is in the time domain and is the result of the practical use of pulse width modulation to approximate electric potential sinusoidal waveforms that need to be applied to each individual microelectrode. The influence of various types of actuating waveforms on particle’s ponderomotive force is studied with a particle-fluid system model in the time-dependent study and which is termed Time Discretization Error study. The following sections present numerical models of said errors and provide analysis of the obtained results in the context of the EPµAM process.

### 1.3. Numerical Studies

In this article, two types of numerical studies are presented. In the first study, a 2D model of the EPμAM process is built in COMSOL Multiphysics software (v5.3, Burlington, MA, USA) [47]. This model consists of two steps. The first step computes the electric fields for six different electrode geometries and for a pre-computed electric potential distribution (10). Then, the electric field solution from the first step is used as an input to the second simulation step where particle trajectories for 21 test-particles are computed. These trajectories are exported from COMSOL and subsequently post-processed in MATLAB where the mean square displacements (MSD) are computed for the test-particle groups. Based on these MSD values, each electrode geometry is evaluated on four metrics relevant to the EPμAM process. These metrics are explained in the comparative matrix subsection of this article. The two top scoring electrode geometries are parametrized and the whole study cycle is repeated on their geometrical variants. Finally, the effects of changing the two electrodes’ geometries are analyzed and reported in terms of the computed MSDs.

The reason for using the mean square displacement technique for analysis of space discretization errors in this article is twofold. First, MSD is a common analysis tool in colloidal science [48,49] and particle groups are the main actors in the EPμAM process. The MSD curves describe the behavior of the system and via fitted equations can quantify the system diffusion and particle mobility coefficients [50]—these coefficients represent physically meaningful and relevant goodness factors of the EPμAM process. Second, the test-particles employed in the mentioned simulations are essentially probes—to draw an analogy with the Lagrangian description of fluid flow—their trajectories essentially describe the constructed electric field in an illustrative way. Because these particles are propelled with electric fields generated via a microelectrode array, the particle trajectory deviations show the effect of the space discretization error and MSD analysis is a convenient way to quantify and analyze their effects.

For the second type of numerical studies, the COMSOL Multiphysics software was used to compute the time series of the electromechanical force acting on a single particle suspended in a fluid. Three different waveforms were used as a boundary condition that produced a time-varying electric field which, in turn, induced the mentioned electromechanical force on the particle. The types of applied waveforms are: sine, simple pulse and computed PWM pulse train waveforms. The sine waveform is commonly used for actuation purposes in biofluidic research [51] and is extensively used in the analytical and theoretical treatment of the dielectrophoresis phenomenon [52]. The pulse waveform represents the simplest implementation for an actuating potential and is commonly used for electrophoretic deposition [4]. The computed PWM waveform represents a practical implementation of a modulated sine wave on the EPμAM device. The obtained results from numerical studies with these waveforms, the electromechanical force time series, and the computed mechanical impulse give both the scope and a way to compare the time discretization error of the used waveforms.

## 2. Methods

### 2.1. Space Discretization Error Studies

**Figure 4:**
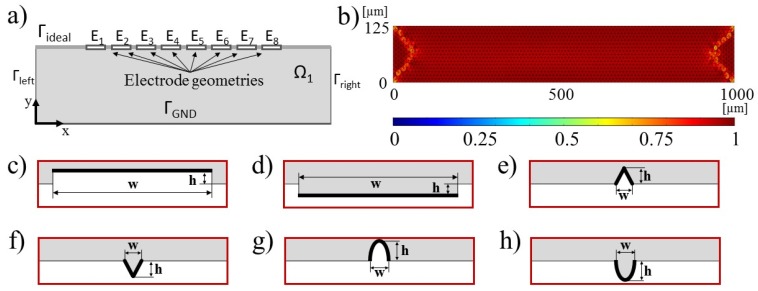
(**a**) Schematic of COMSOL SDE model; (**b**) Generated mesh for ideal electric potential boundary condition (BC); (**c**) Step electrode; (**d**) Channel electrode; (**e**) Pin electrode; (**f**) Notch electrode; (**g**) Parabolic pin electrode; (**h**) Parabolic notch electrode.

To account for the electrode shapes and sizes a 2D model of the EPμAM apparatus was built in the COMSOL Multiphysics v5.3 software (Figure 4a). In this model, we used two steps: (i) computation of the electric fields of the fluid domain for the given boundary conditions and afterward, (ii) computation of the test particles’ trajectories in the computed electric fields. The computed trajectories are further processed via mean square displacement analysis and compared to the test particle’s trajectories from the ideal electric potential distribution, which was also implemented in COMSOL. Figure 4b shows the generated triangle mesh, where the colored scale defines the skewness quality of the generated FEM elements, with the value of 1 indicating a perfect isotropic element [53].

For this study, we selected several types of electrode shapes, based on common usage and/or ease of fabrication. These shapes are Step, Channel, Pin, Notch, Parabolic pin and Parabolic notch. They are all shown in Figure 4c–h. Note that the electrode geometries in the 2D model (Figure 4a) are flipped horizontally/rotated by 180°.

In these figures, grayed out areas correspond to the computational fluid domain Ω_1_, whereas the white parts are outside of the numerical model. The electrodes (E_1_–E_8_) used in the simulations are of the same shape, no combination of electrode shapes was considered during this analysis. All shapes were defined with two parameters, width *w* and height *h*. For the Parabolic pin and Parabolic notch geometries, the width and the height parameters correspond to the elliptical (semi)axis lengths. The geometry parameters *w* and *h* used to define electrode geometries are shown in Table 1. The computed electric potential was applied as the boundary condition (BC) across the thick black lines, shown in Figure 4c–h. For the COMSOL simulations, we have considered gravity, drag and dielectrophoretic forces by enabling their computation across the whole simulation domain (Ω_1_).

**Figure 5:**
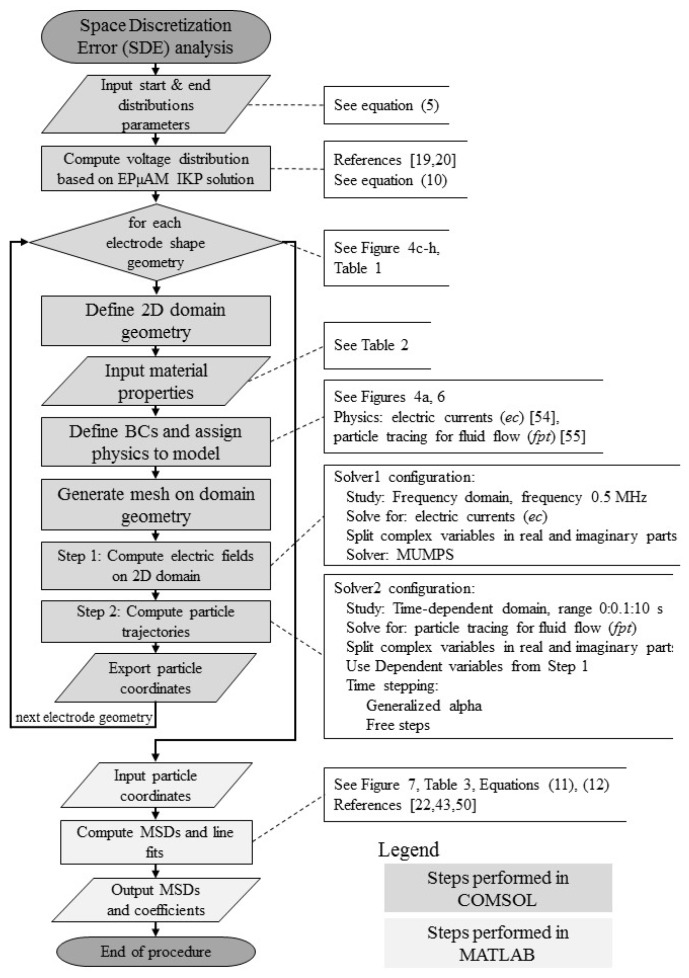
Space discretization error analysis flowchart.

The developed space discretization error analysis is depicted in the Figure 5 flowchart alongside all model and solver configuration parameters that we changed from default COMSOL values. The reader is referred to COMSOL manual for implementation details and model building of electric currents physics [54] (p. 140) and particle tracing physics [55]. After defining each new electrode geometry, the domain was re-meshed with free triangular elements. Due to the small size of electrode geometries, relative to the size of the computational domain, the obtained meshes did not deviate much from the mesh shown in Figure 4b.

**Figure 6:**
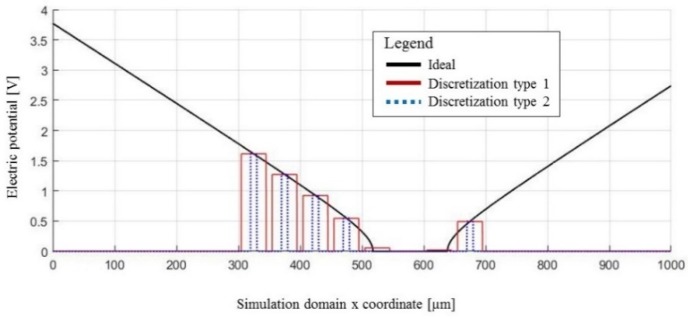
Computed electric potential values used for the top boundary condition.

The applied boundary conditions to the top boundary of the COMSOL model are shown in Figure 6.

The ideal potential distribution (Figure 6, Ideal) was applied across the whole top boundary (depicted as a thick gray line in Figure 4a). The wider bar-like potential distribution (Figure 6, type 1) was used with the Step and Channel electrode types (Figure 4c,d). The narrower, bar-like potential distribution (Figure 6, type 2) was used with the remaining electrode geometries: Pin, Notch, Parabolic pin and Parabolic notch (Figure 4e–h). This potential was applied with the special *aveop1*() COMSOL operator [56]. This operator computes the average values of the ideal electric potential distribution for the given electrode surface geometry and essentially functions as a discretization scheme. These average values are shown in Figure 6, alongside the ideal electric potential distribution. The electric potential, shown in this image, is supposed to move the initial particle distribution with a mean of 500 µm and standard deviation of 50 µm to the final distribution that has a mean of 550 µm and a standard deviation of 30 µm across the x coordinate of the 2D model.

**Figure 7:**
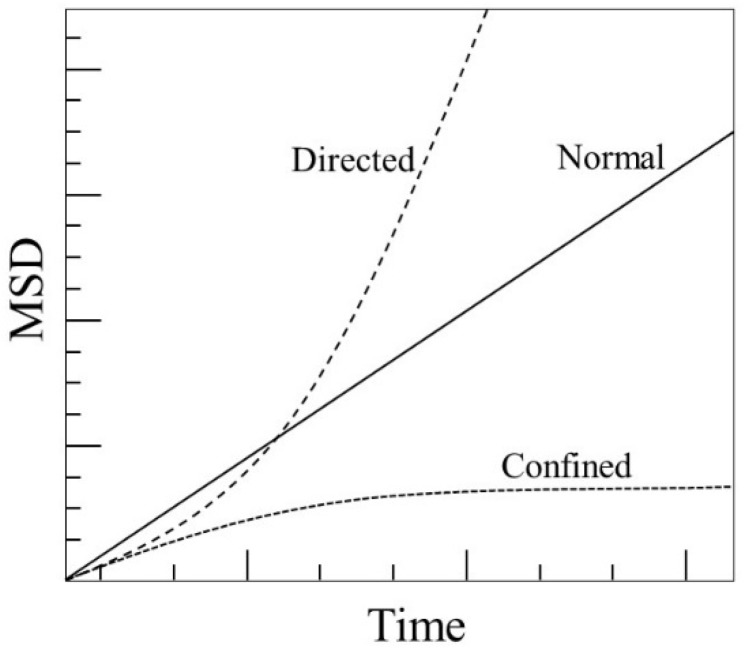
Example of mean square displacement vs. time plots showing examples of normal, directed and confined diffusions for a particle ensemble. Adapted from Reference [50].

The particle trajectories solution obtained in the final step of the COMSOL simulation was exported and post-processed in MATLAB with MSD analysis. Mean square displacement analysis is a commonly used technique in colloidal studies to determine the modes of particle displacement under different conditions [22,23]. Illustrations of the normal, confined, and directed modes of motion are shown in Figure 7.

In the context of the EPμAM process, this technique is being used to estimate the diffusion of the test particle groups as well as their enhanced mobilities from the application of the electric fields. The linear fit corresponds to the systems where normal, non-directed diffusion is dominating the motion:

(11) $$\langle r^{2}\rangle =2dDt$$

The quadratic fit corresponds to the systems where external forces have a significant influence on the particle ensemble:

(12) $$\langle r^{2}\rangle =2dDt+v^{2}t^{2}$$

In (11) and (12), *r* is the particle displacement vector, and 〈…〉 represents averaging over all particles in the ensemble, *D* is the diffusion coefficient, *v* is particle mobility and *t* is time. Factor *d* represents the dimensionality of the problem (i.e., 2 for two-dimensional problem/model). Based on Einstein’s analysis [57], for diffusion under normal circumstances, the computed MSD has a linear relationship to time. If the particles in question are confined in some sort of potential trap during the observational time, then the MSD would trail off and plateau at a certain time, as depicted in Figure 7. If the particles are under the influence of additional forces then their MSD will be higher than in the normal case and will not have a linear relationship with respect to the observation time. Equations (11) and (12) were fitted to the computed MSD curves in MATLAB post-processing step.

After the MSD analysis, the electrode shape geometries were graded within a comparative evaluation matrix and the top two candidates were further analyzed as case studies. In these case studies, the two selected electrode geometries were varied and MSD for the whole particle ensemble was recomputed.

### 2.2. Analysis of Time Discretization Errors

**Figure 8:**
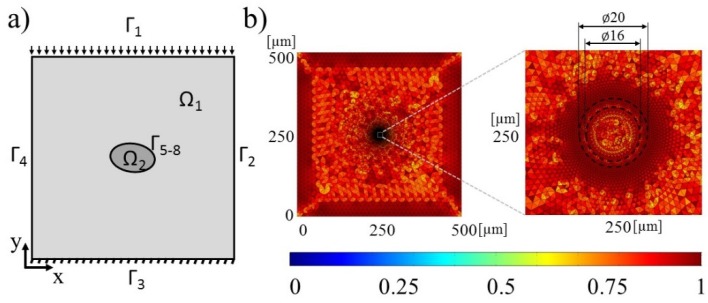
(**a**) Schematic of COMSOL TDE model; (**b**) Generated mesh (**left**) with the expanded detail of generated mesh in the particle’s vicinity (**right**).

To compare particle actuation with sine and pulse waveforms, a 2D numerical model is designed and built within the COMSOL suite (Figure 8). This 2D model represents a single particle located near the center of the domain with appropriate material properties (Ω_2_) and surrounded by a fluid, which has different electrical properties (Ω_1_) (see Table 2). The bottom edge of the domain (Γ_3_) is set as the ground electrode (Dirichlet boundary condition, Γ_3_ = 0 (V)) while the top edge of the domain (Γ_1_) is actuated with the waveforms. The particle surface is defined with subdomain boundaries (Γ_5_ to Γ_8_). The material properties of the particle and the fluid medium are set to be that of alumina, and water, respectively. To improve mesh quality around the particle (20 µm diameter circle), an inner shell with a 16 µm diameter was added inside the particle to help guide the automatic meshing of the 2D model (Figure 8b). The color bar legend in Figure 8b represents the skewness element quality.

**Figure 9:**
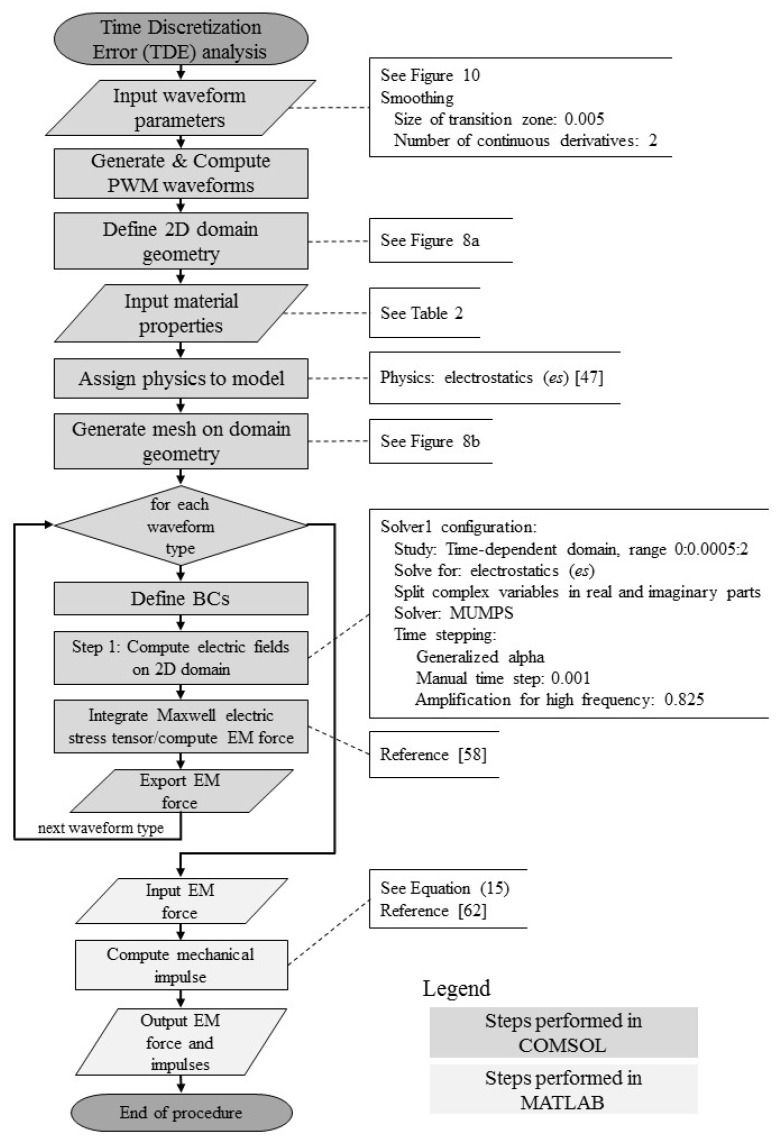
Time discretization error analysis flowchart.

**Figure 10:**
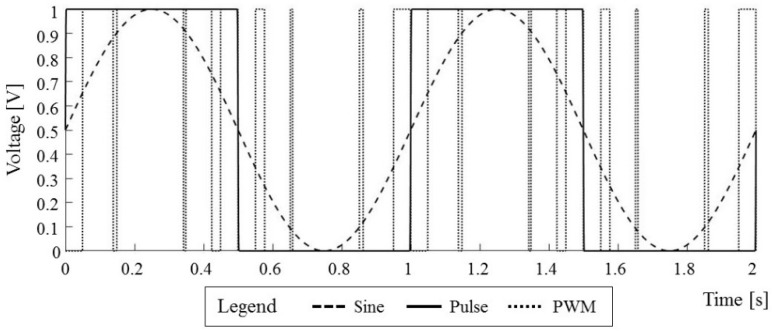
Top boundary condition waveforms.

The implemented procedure for the time discretization error analysis is shown in the flowchart in Figure 9. All simulations had the same setup—the only part that was changed was the top boundary condition (Figure 8a, Γ_1_ boundary) as the sine, pulse, and PWM waveforms. The applied waveforms are shown in Figure 10; they all had a unit amplitude and frequency and were applied over two periods. After the simulations completed, the time series of the computed electromechanical (EM) force were exported and post-processed in MATLAB.

The source of the EM force stems from the uneven potential distribution on the particle’s surface. This uneven potential distribution comes from the polarization of the particle surface due to the applied electric potential waveform. The EM force can be calculated by integrating the Maxwell stress tensor [42] and [54] (p. 65):

(13) $$\overset{=}{T}=\varepsilon \bar{E}\bar{E}-0.5\overset{=}{I}\left(\varepsilon \bar{E}\cdot \bar{E}\right)$$

where $\overset{=}{T}$ is the Maxwell stress tensor in the absence of magnetic fields, *ε* is the permittivity of the medium (in this case of the fluid), $\bar{E}$ is the electric field and $\overset{=}{I}$ is the identity tensor. When this tensor is integrated over the outer surface of the particle (defined here with an outward unit facing normal vector $\hat{n}$) one obtains the total electromechanical force, $\bar{F}$_EM_, on the enclosed surface *S*:

(14) $${\bar{F}}_{EM}=\int_{S}\left(\overset{=}{T}\cdot \hat{n}\right)dA$$

Double overbars are used to explicitly delineate the difference between the vectors and higher-order tensors. Calculating the electromechanical force via the Maxwell stress tensor surface integration is the most general method of obtaining the forces on the particles. It does not depend on the particle’s shape, such as some of the analytical solutions [52] but it is computationally expensive. This approach is adopted in the current numerical studies because it allows the computation of the electromechanical force for arbitrary waveforms and not just sinusoidal ones. This electromechanical force, in the case of an uncharged particle, is an analog to the dielectrophoretic force and its multipolar expansion [58,59].

The obtained EM force time series were numerically integrated in MATLAB [60] with the *trapz*() function. From the basic principles of mechanics [61], the time integral of force *F* is equal to the mechanical impulse *J*, which can be rewritten as a change in linear momentum *p_i_*. In the case that the particle, or object in general, has a constant mass, this change of linear momentum describes a change in particle velocity, i.e.,

(15) $$J=\int_{t_{1}}^{t_{2}}Fdt=\int_{p_{1}}^{p_{2}}dp=p_{2}-p_{1}=m\left(v_{2}-v_{1}\right)$$

This mechanical impulse *J* represents the accumulated pondermotive force effects applied on a particle during one, or more, oscillation periods and can be treated as a comparison basis for the different waveforms applied in terms of waveform potency to actuate a particle.

## 3. Results and Discussion

In this section, the numerical results from the time- and space-discretization analysis are presented. For the space-discretization analysis, the computed particle trajectories for a given electrode shape variations are shown first, followed by a geometric variation study of the top two electrode geometries. For the time-discretization analysis, the exerted electromechanical forces on the particle are calculated and presented in the form of time series and computed mechanical impulses.

### 3.1. Space-Discretization Error Results

**Figure 11:**
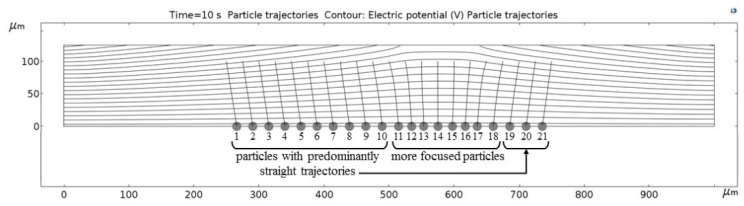
Computed particle trajectories and electric potential for the ideal electric potential BC.

**Figure 12:**
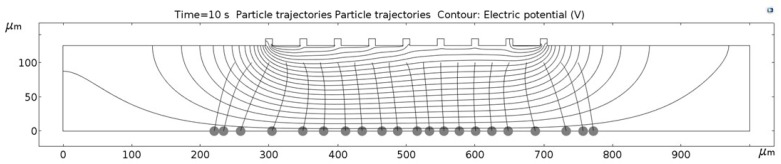
Computed particle trajectories and electric potential for the Step electrode geometry.

**Figure 13:**
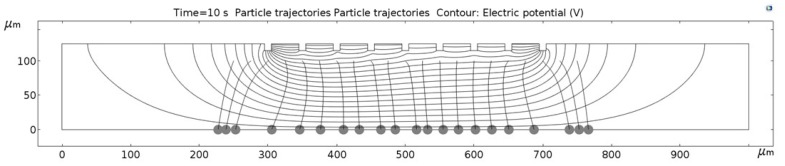
Computed particle trajectories and electric potential for the Channel electrode geometry.

**Figure 14:**
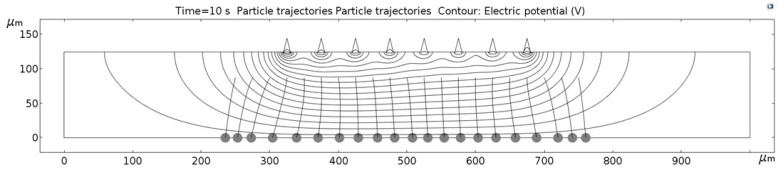
Computed particle trajectories and electric potential for the Notch electrode geometry.

**Figure 15:**
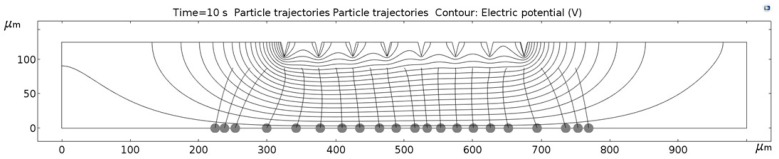
Computed particle trajectories and electric potential for the Pin electrode geometry.

**Figure 16:**
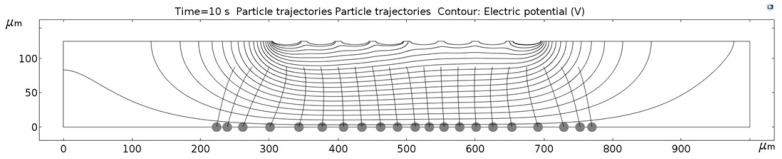
Computed particle trajectories and electric potential for the Parabolic pin electrode geometry.

**Figure 17:**
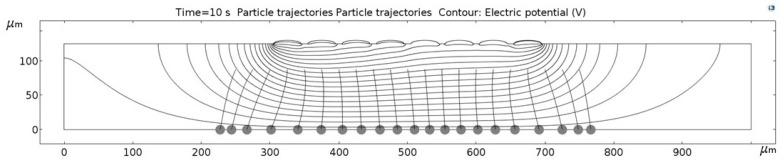
Computed particle trajectories and electric potential for the Parabolic notch electrode geometry.

Figure 11, Figure 12, Figure 13, Figure 14, Figure 15, Figure 16 and Figure 17 show the computed trajectories for the ideal and discretized cases of the applied electric potential. Each simulation uses 21 test particles (gray circles) that are released from a uniform grid at the beginning of the simulation. Black contours are electric equipotential lines, computed in the first simulation step. Each figure has 20 equidistant potential contour plots, spanning the range from the maximum electric potential (see Figure 6) down to electric ground, set at −5 V. The trailing black lines behind the test particles are their computed trajectories, computed in the second simulation step (for details refer to the flowchart in Figure 5). This 2D simulation is modeling a single application cycle of the computed electric potential, whereas in the actual EPμAM process the computed electric potential would be sequentially applied until the particles are grouped at the desired location.

In the case of the ideal electric potential (Figure 11), the test particle trajectories are predominantly straight and some are generally focused around the desired region (around 520–580 µm on the horizontal axis). For all the other cases (Figure 12, Figure 13, Figure 14, Figure 15, Figure 16 and Figure 17), a notable difference is observed. Namely, the leftmost and rightmost three particles have deviating trajectories and these particles tend to form subgroups when deposited on the bottom edge of the model.

First, a qualitative comparison between the test particle trajectories for the ideal electric potential distribution on one and distributions for the employed six electrode configurations on the other side shows the biggest difference for the three test particles on the edges of the ensemble. This difference can be explained by the variations in the computed electric fields and the electric potential distribution, which are shown in Figure 11, Figure 12, Figure 13, Figure 14, Figure 15, Figure 16 and Figure 17 as black contour plots. For the ideal case, the applied BC spans across the whole top edge of the model domain, whereas, for the electrode shapes it is applied only across the narrow electrode surfaces and that in the averaged form (see discretization scheme types 1 and 2 in Figure 6). This diverging electric potential distribution is the main cause for the defocusing of the test particle ensembles. For the EPμAM apparatus, this means that deposition near the edges of the microelectrode array should be avoided due to the lack of accuracy and the fringing effects of the diverging electric fields.

**Figure 18:**
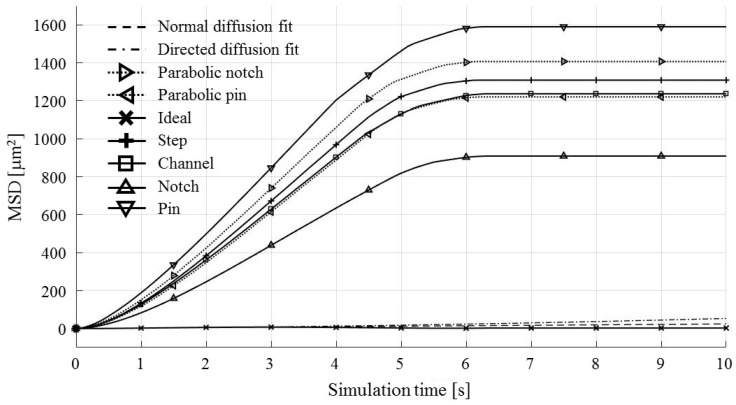
MSD curves for test particles 1–7 (left third).

**Figure 19:**
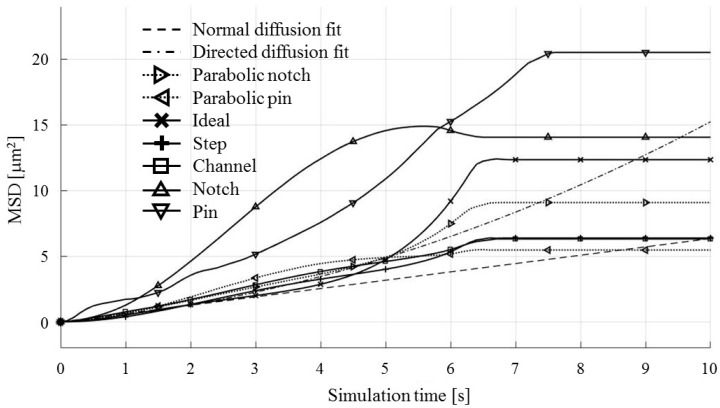
MSD curves for test particles 8–15 (middle third).

**Figure 20:**
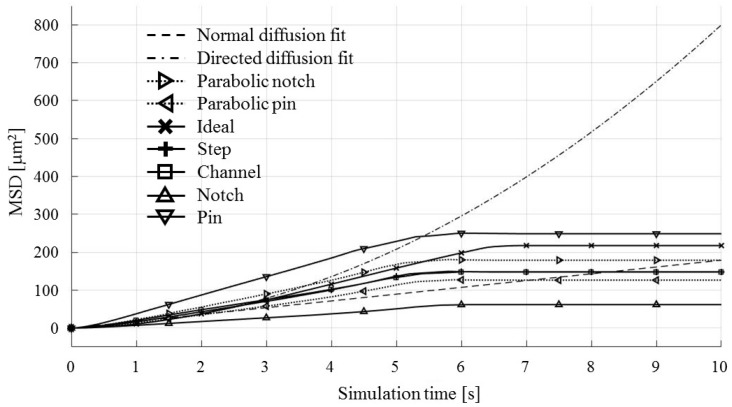
MSD curves for test particles 16–21 (right third).

Each test particle was labeled from left to right with numbers from 1 to 21 (see the bottom of Figure 11). The x coordinates of 21 particles were evenly distributed between 250 and 750 µm in 25 µm increments. The y coordinates of the particles were set as 87.5 µm. The MSD analysis was then subsequently performed on left (particles 1–7), middle (particles 8–15) and right (particles 16–21) third of the whole particle ensemble. Figure 18, Figure 19 and Figure 20 show the mean square displacement curves of these three subgroups. Each figure also has linear and quadratic fitted curves (11) and (12) on the first quarter of the time series for the ideal particle case, which is used to estimate particle diffusion coefficients and mobilities [50]. In all the figures, most MSD curves level off after 7 s of simulation time, indicating that the particle ensembles have stopped moving/have been deposited.

The electrode shape MSDs have similar trends in general—the differences primarily lie in the ramps’ slopes (MSD values between 0 and 6 s of simulation time). They do not have the shape of directed, but of normal and confined MSD types (Figure 7).

For the case of the middle third of the particle ensemble (Figure 19), the MSD curves show the smallest spread of the MSD values. The nonlinearities, observed for some types of electrode shapes, are in the form of ramp-like increases in the computed MSD (e.g., Notch and Parabolic notch MSDs).

The results for the rightmost third of the particle ensemble, depicted in Figure 20, show the smallest difference of all three plots (Figure 18, Figure 19 and Figure 20) with respect to the shapes of the computed MSD values. The MSD of the Pin electrode configuration is the only one configuration which has higher computed MSD values than the ideal MSD ones, over all simulation time intervals.

Table 3 shows the computed values of the ensemble’s diffusion coefficients D_L__*_, D_Q__*_ for linear and quadratic fits (11) and (12), respectively, as well as particle ensemble mobilities v_Q__*_, derived from the quadratic curve fits (* stands for *L*—left, *M*—middle, *R*—right thirds).

Overall, the smallest values are computed for the middle third of the test particle ensembles. The diffusion coefficients, computed for the linear and quadratic fits on the middle third of the ensemble, are relatively close in values, which should suggest that the test particle movement is mostly diffusion dominated. This could also be inferred from the shapes of the MSD curves in Figure 19 and by comparing them with the curves in Figure 7. On the other hand, the largest computed differences are between the diffusion coefficients for the linear and quadratic fits of the right third test particle ensemble. The spuriously high values of the linear fit coefficient (*D_LR_*) can partially be accounted for by considering both coefficients of the analogous quadratic fit (*D_QR_* and *v_QR_*).

The computed values in Table 3 can be related to the EPμAM process in the following manner. The applied electric potential on the microelectrode array is to: (i) Slightly group the particle ensemble and (ii) push the whole ensemble in the right (+x direction in the model coordinate system) direction (Figure 6). This grouping action can be inferred by comparing the computed coefficients in Table 3 with the intended functions (i and ii) of the applied electric potential. The middle third of the particle ensemble has the smallest diffusion coefficients because the particles are grouping, as intended, in this region. Second, the smallest diffusion coefficient values are for the leftmost third of the particle ensemble where the effects of grouping alongside with pushing the whole ensemble to the right constructively interact. Lastly, the rightmost particle ensemble has the highest values of the diffusion coefficients and mobilities due to the predominant effect of the pushing action of the applied electric potential, which destructively interacts with the focusing/grouping effect of the applied electric potential, and causes particles to overshoot and spread further apart.

### 3.2. Comparative Evaluation Matrix

To select suitable electrode shape candidates for more in-depth analysis, a comparative evaluation matrix for all six electrode shape primitives is constructed (Table 4). This evaluation matrix uses four metrics: (i) difference between the computed MSDs and the MSD of the ideal case; (ii) whether the observed time interval of the MSD change is larger than the interval for the ideal case; (iii) whether the computed MSDs are between the linear and quadratic fits of the ideal MSD and (iv) which of the computed MSDs have values closest to the ideal case before the MSD value reaches its plateau (~6 s in simulation time). These metrics are applied to the three sub-ensembles (left, middle and right thirds of the 21 test particles) and then averaged across the whole particle ensemble.

The purpose of the first metric is to determine which electrode shape has the highest potential to combat the particle diffusion process by the application of the strongest electric potentials. From the process control standpoint, it is always possible to decrease the applied electric potential to prevent particle overshooting or electrode surface degradation. The second metric ensures that, for a given electrode shape, the test particle ensemble stays under the influence of the nonuniform electric field for the longest viable time—this ensures that the cyclical application of the electric potential distribution has the highest efficiency. The third metric checks that the imbued electrophoretic mobility of the electrode shape in question can be reasonably approximated with diffusion and mobility coefficients from the MSD analysis, which may simplify the implementation of the control system/loop. The purpose of the fourth metric is to determine the electrode shape that would have the smallest energy losses which would improve the EPμAM device’s energy efficiency in the long run.

As a final score, an average and weighted average are computed. The weighted average score function is to account for (i) decreased efficiency of electrode shapes to function on the edges of the microelectrode array and for (ii) the predominant role of the middle array electrodes in the deposition process. This is accomplished by multiplying the middle sub ensemble score by a factor of two, summing it with the left and right sub ensembles and dividing the obtained sum by four. The results are presented in Table 4.

Based on the computed scores in Table 4, the Pin and Parabolic notch electrode shapes have the highest and second highest values in both scoring cases, respectively. These two electrode geometries will be more thoroughly reevaluated in the following case study subsection.

### 3.3. Parametric Sweeps of Pin and Parabolic Notch Electrode Geometries—Case Studies

In this subsection, a more in-depth evaluation of the Pin and Parabolic notch electrode geometries is presented. For each electrode shape, a partial parametric sweep of the electrode widths and heights (defined in Figure 4e,h) is performed and the same MSD analysis is subsequently performed on the electrode shape variants.

**Figure 21:**
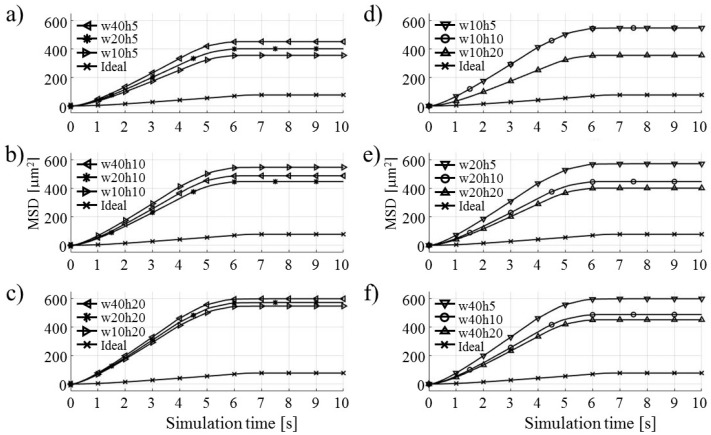
Computed MSD values for Pin electrode shape geometry variations: (**a**–**c**) varying width *w* only: 10, 20, 40 (µm); (**d**–**f**) varying height *h* only: 5, 10, 20 (µm).

Figure 21 shows the computed MSD for parametric sweeps of the Pin electrode geometry. The ideal electric potential distribution MSD values are plotted for reference purposes.

In Figure 21a–c, the height of the electrode was held constant at three levels 5, 10 and 20 µm (Figure 21a, b, and c respectively), while the width of the electrode was set to 10, 20, and 40 µm. The increase in the width of the electrode increased the computed MSD values, which can be explained by the increased electric flux coming from the increased overall area of the electrode. However, in Figure 21b, this trend was not observed—the highest MSD was obtained for the smallest electrode width of 10 µm. A possible explanation for this result may be that this specific electrode geometry experiences some form of nonlinearity or this may be the result of the statistical nature of MSD analysis. This case may warrant further, closer examination. In Figure 21d–f, the width of the Pin electrode geometry was held constant at 10, 20, and 40 µm (Figure 21d, e, and f respectively) and the height of the electrode was varied as 5, 10, and 20 µm. For these cases, the electrode height increase caused an increase in the computed MSD values. For the initial case of the 10 µm electrode width, the values of MSD for 10 and 20 µm height overlapped. This could be related to the observed nonlinearity in Figure 21b. In general, for the Pin electrode geometry, the increase of both parameters—width and height—increased the computed MSD values.

**Figure 22:**
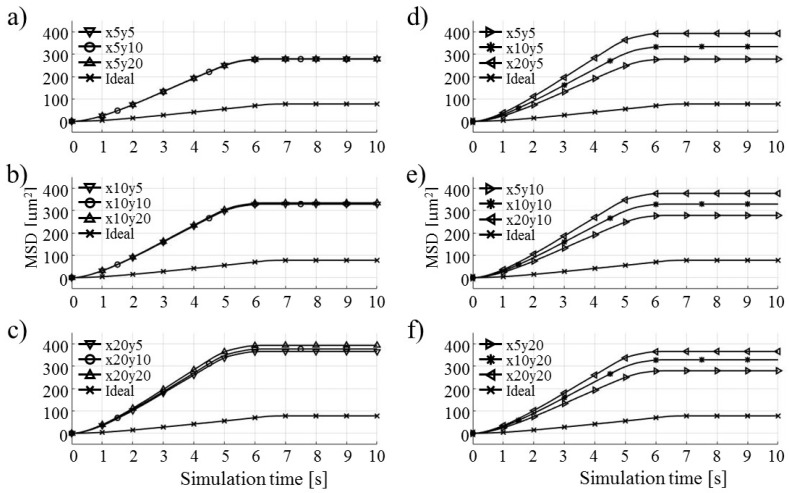
Computed MSD values for parabolic notch electrode shape geometry variations: (**a**–**c**) varying only half-height y (0.5*h*): 5, 10, 20 (µm); (**d**–**f**) varying only half-width x (0.5*w*): 5, 10, 20 (µm).

Results of parametric sweeps for the Parabolic notch electrode geometry are shown in Figure 22. In Figure 22a–c, the half-width of the Parabolic notch geometry was held constant at 5, 10, and 20 µm levels (Figure 22a, b, and c respectively) while the electrode half-height was varied from 5, 10, to 20 µm values. The figures show that for small values of the half-width, all computed MSDs overlap, which indicates a small influence of the Parabolic notch height on particle MSD computations. Only in the case of a 20 µm half-width is it observed that the half-height increase causes a decrease in the computed MSD. Figure 22d–f shows the variations for electrode half widths of 5, 10, and 20 µm while holding a constant electrode half-height at 5, 10, and 20 µm values (Figure 22d, e, and f respectively). For all three cases, an increase in the electrode half-widths causes increases in the computed MSD for the test particle ensemble. Overall, for the Parabolic notch electrode geometry, the half-width parameter plays a more important role than the half-height parameter.

### 3.4. Time-Discretization Error

**Figure 23:**
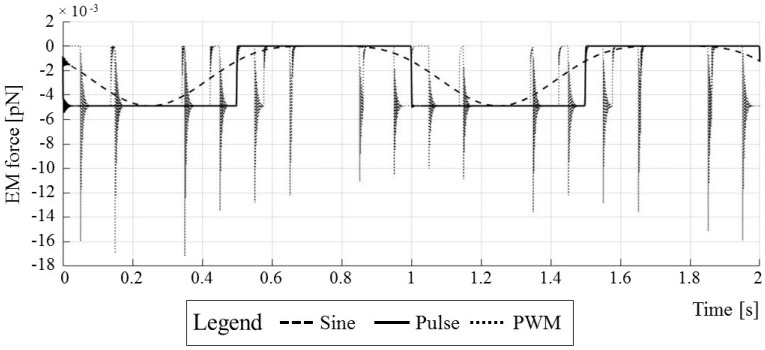
Time discretization error study results.

A qualitative comparison between the particle EM forces, computed for different applied boundary conditions (e.g., Sine, Pulse, and PWM waveforms) is shown in Figure 23.

The computed EM force magnitude of the PWM waveform is of the same order of magnitude as for the sinusoidal and rectangular pulse waveforms. Negative values in Figure 23 stem from the computed EM force direction opposite to the y-axis direction of the adopted coordinate system in the COMSOL TDE model (see Figure 8a). The calculated force time series for the PWM waveforms and actuating PWM waveforms have the same trends, barring (i) oscillations on the rising pulse edges and (ii) trail-offs on the dropping edges.

The mechanical impulses *J* of waveforms were computed by integrating EM forces in time. This was accomplished by exporting EM forces from the COMSOL simulations and evaluating them with the discretized trapezoidal rule in MATLAB. The mechanical impulse values for the Sine, Pulse and PWM waveforms are 0.0037, 0.0047, and 0.0050 (pN·s) respectively. The Pulse and PWM mechanical impulses, normalized with the Sine mechanical impulse, are 1.2759 and 1.3711. The Pulse waveform applies ~27% more impulse to the particle, whereas a PWM waveform applies ~37% more with respect to the Sine waveform baseline. According to (15), this translates into increased particle mobility when Pulse (27% increase) and PWM (37% increase) waveforms are used.

### 3.5. Discussion

This paper presented a use of multiphysics FEA studies to study and quantify space and time errors in a micro additive manufacturing process which is based on the deposition of particles from colloidal solutions. Several appropriate microelectrode geometries were studied and compared via the mean square displacement method (MSD), commonly used in colloidal science. The computed MSD curves showed increased values of diffusion and mobility coefficients when compared to the ideal electric potential case. For all electrode cases, the deposited particle positions were scattered across the ground electrode—only for the ideal electric potential case the deposited particles were partially grouped on the ground electrode. Because fluid flow was not included in this simplified process model it would not be possible to specifically comment on the significance of this trajectory deviation and the scattering of the deposited particles. Moreover, the EPμAM process model used in the space-discretization error study simulated only a single deposition cycle—in the real process, multiple cycles would be used to gradually deposit particles in the desired region.

The parametric study of the two top microelectrode candidates—pin and parabolic notch geometries—demonstrated the influence of electrode sizes and shapes on particle trajectories which is nonlinear in nature. In general, bigger electrode size directly translated into larger electric flux which increased the mobilities of nearby particles; electrode shapes (i.e., convex or concave geometry) had secondary effects on the computed MSDs.

The computed mechanical impulses in the TDE studies have shown that, although the magnitude of the applied EM force on the particle does not change with different waveforms, their cumulative effects in time do show a significant difference. This can be exploited for faster deposition but should also be accounted for in cases of higher accuracy applications.

The numerical results have shown that electric field modulation has an influence on particle diffusion phenomena at this process scale. It would be conceivable to apply the same control mechanism, not to deposit particles but to initiate or partially govern chemical processes, which would open another application avenue for the EPμAM process. However, careful model calibration with particle deposition experiments will be necessary before this approach is explored.

## 4. Conclusions and Future Work

Two types of multiphysics FEM studies were presented and used to qualitatively and quantitatively estimate test particle trajectory errors within a new micro additive manufacturing process—EPμAM:

- The space discretization study assessed the computed particle trajectories using the MSD technique. The obtained MSD curves allowed the selection of the top microelectrode geometry candidate for the EPμAM process. Partial geometric sweep FEM studies of the top two (out of six) electrode geometries demonstrated the nonlinear influence of the electrode geometry on particle mobilities.
- The time discretization study computed the time series of the electromechanical force (for three given actuating waveforms) acting on spherical particles in the middle of the EPμAM device’s workspace. The Pulse and PWM waveforms outperformed the Sinusoidal waveform with a 27% and 37% increase, respectively, in particle mobility.

One of the future steps could start from the observed nonlinear behavior of electrode geometries and perform optimization or sensitivity studies under more specific process or material requirements. On this scale, electrode-fluid interactions and consequent electrochemical processes should be incorporated into the model.

The natural expansion of this work would be to incorporate multiple deposition cycles in the simulations in which the intermediate particle distribution after the current deposition cycle would be used as an input to the EPμAM control algorithm for the next deposition cycle.

The simple EM force model could be expanded by coupling fluid mechanics effects to enable the change of particle position and the tracking of particle mobility across a greater range. Variable particle sizes and shapes should also be considered in the next steps.

## Figures and Tables

**Table 1:** Electrode Geometry Parameters.

Electrode Geometry	Width, *w* (µm)	Height, *h* (µm)
**Step**	40	10
**Channel**	40	10
**Pin**	10	20
**Notch**	10	20
**Parabolic pin**	40	10
**Parabolic notch**	40	10

**Table 2:** Material Properties used in the single particle 2D model.

Material domain	Ω_1_: Water (H_2_O)	Ω_2_: Alumina (Al_2_O_3_)
**Relative permittivity (-/-)**	80.1	9.9
**Electrical conductivity (S/m)**	2.5 × 10^−2^	1 × 10^−13^
**Density (kg/m^3^)**	1000	2000
**Dynamic viscosity (Pa·s)**	1 × 10^−3^	Not Applicable

**Table 3:** Computed coefficients for the linear and quadratic fits of the MSD Equations (11) and (12).

Coefficient		Confidence Bounds (95%)	
Name	Value	Lower	Upper
***D_LL_***	0.607	0.582	0.632
***D_LM_***	0.159	0.152	0.165
***D_LR_***	4.472	4.082	4.865
***D_QL_***	0.436	0.365	0.507
***D_QM_***	0.106	0.092	0.120
***D_QR_***	0.806	0.666	0.946
***v_QL_***	0.598	0.462	0.708
***v_QM_***	0.332	0.287	0.371
***v_QR_***	2.770	2.718	2.821

**Table 4:** Comparative evaluation matrix of the electrode shapes’ MSD analysis.

Electrode Shapes		Metric				Scores		
Importance (1–4) 4—Highest 1—Lowest		MSD > MSD_*ideal*_	*dt* > *dt_ideal_*	Between Fits	Closest to Ideal	Subgroup Scores	Average Score	Weighted Average
Importance →		3	4	2	1			
Step	L	4	2	0	3	23	14.00	13.50
	M	1	0	2	5	12		
	R	0	0	1	5	7		
Channel	L	3	2	0	4	21	14.67	14.50
	M	2	0	2	4	14		
	R	0	0	2	5	9		
Notch	L	1	2	0	6	17	14.33	17.00
	M	6	1	1	1	25		
	R	0	0	0	1	1		
Pin	L	6	2	0	1	27	26.67	26.75
	M	5	2	1	2	27		
	R	6	1	1	2	26		
Parabolic pin	L	3	2	0	4	21	14.67	14.75
	M	2	1	1	3	15		
	R	0	0	2	4	8		
Parabolic notch	L	5	2	0	2	25	19.00	18.00
	M	1	1	1	6	15		
	R	3	0	1	6	17		

## Supplementary Files

Supplementary File 1

## References

[1] Velev O.D., Bhatt K.H. (2006). On-chip micromanipulation and assembly of colloidal particles by electric fields. Soft Matter.

[2] Corni I., Ryan M.P., Boccaccini A.R. (2008). Electrophoretic deposition: From traditional ceramics to nanotechnology. J. Eur. Ceram. Soc..

[3] Khoshmanesh K., Nahavandi S., Baratchi S., Mitchell A., Kalantar-zadeh K. (2011). Dielectrophoretic platforms for bio-microfluidic systems. Biosens. Bioelectr..

[4] Ammam M. (2012). Electrophoretic deposition under modulated electric fields: A review. RSC Adv..

[5] Wereley S.T., Meinhart C.D. (2010). Recent advances in micro-particle image velocimetry. Annu. Rev. Fluid Mech..

[6] Lee S.J., Kim S. (2009). Advanced particle-based velocimetry techniques for microscale flows. Microfluid. Nanofluid..

[7] Peterson S.D., Chuang H.S., Wereley S.T. (2008). Three-dimensional particle tracking using micro-particle image velocimetry hardware. Meas. Sci. Technol..

[8] Virant M., Dracos T. (1997). 3D PTV and its application on Lagrangian motion. Meas. Sci. Technol..

[9] Oddy M.H., Santiago J.G. (2004). A method for determining electrophoretic and electroosmotic mobilities using AC and DC electric field particle displacements. J. Colloid Interface Sci..

[10] Meinhart C.D., Wereley S.T., Gray M.H.B. (2000). Volume illumination for two-dimensional particle image velocimetry. Meas. Sci. Technol..

[11] Lindken R., Rossi M., Große S., Westerweel J. (2009). Micro-particle image velocimetry (µPIV): Recent developments, applications, and guidelines. Lab Chip.

[12] Westerweel J. (1997). Fundamentals of digital particle image velocimetry. Meas. Sci. Technol..

[13] Huang H., Dabiri D., Gharib M. (1997). On errors of digital particle image velocimetry. Meas. Sci. Technol..

[14] Paprotny I., Doering F., White R.M. MEMS particulate matter (PM) monitor for cellular deployment. Proceedings of the 2010 IEEE Sensors.

[15] Chakrabarty K., Zeng J. (2005). Design automation for microfluidics-based biochips. ACM J. Emerg. Technol. Comput. Syst. (JETC).

[16] Wang X., Chen S., Kong M., Wang Z., Costa K.D., Li R.A., Sun D. (2011). Enhanced cell sorting and manipulation with combined optical tweezer and microfluidic chip technologies. Lab Chip.

[17] Prakash P., Deng G., Converse M.C., Webster J.G., Mahvi D.M., Ferris M.C. (2008). Design optimization of a robust sleeve antenna for hepatic microwave ablation. Phys. Med. Biol..

[18] Kataja J., Nikoskinen K. (2011). The parametric optimization of wire dipole antennas. IEEE Trans. Antennas Propag..

[19] Pritchet D., Ehmann K., Cao J., Huang J. Additive Micro Manufacturing with Modulated Electric Fields: Design Challenges and Application Potential. Proceedings of the 11th International Conference on Micro Manufacturing.

[20] Pritchet D., Ehmann K., Cao J., Huang J. Boltzmann law-based control of localized electrophoretic particle deposition and manipulation. Proceedings of the 1st International Conference on Manipulation, Automation and Robotics at Small Scales (MARSS 2016).

[21] Trau M.D.A.S., Saville D.A., Aksay I.A. (1996). Field-induced layering of colloidal crystals. Science.

[22] Michalet X. (2010). Mean square displacement analysis of single-particle trajectories with localization error: Brownian motion in an isotropic medium. Phys. Rev. E.

[23] Duan W., Ibele M., Liu R., Sen A. (2012). Motion analysis of light-powered autonomous silver chloride nanomotors. Eur. Phys. J. E.

[24] Lucena D., Tkachenko D.V., Nelissen K., Misko V.R., Ferreira W.P., Farias G.A., Peeters F.M. (2012). Transition from single-file to two-dimensional diffusion of interacting particles in a quasi-one-dimensional channel. Phys. Rev. E.

[25] Mojarad N., Krishnan M. (2012). Measuring the size and charge of single nanoscale objects in solution using an electrostatic fluidic trap. Nat. Nanotechnol..

[26] Schmidle H., Jäger S., Hall C.K., Velev O.D., Klapp S.H.L. (2013). Two-dimensional colloidal networks induced by a uni-axial external field. Soft Matter.

[27] Dunderdale G., Ebbens S., Fairclough P., Howse J. (2012). Importance of particle tracking and calculating the mean-squared displacement in distinguishing nanopropulsion from other processes. Langmuir.

[28] Lavrentovich O.D. (2014). Transport of particles in liquid crystals. Soft Matter.

[29] Pohl H.A., Crane J.S. (1971). Dielectrophoresis of cells. Biophys. J..

[30] Wang X.-B., Huang Y., Gascoyne P.R.C., Becker F.F. (1997). Dielectrophoretic manipulation of particles. IEEE Trans. Ind. Appl..

[31] Kua C.H., Lam Y.C., Yang C., Youcef-Toumi K. Review of Bio-Particle Manipulation Using Dielectrophoresis. https://dspace.mit.edu/handle/1721.1/7464.

[32] Cummings E.B., Singh A.K. (2000). Dielectrophoretic trapping without embedded electrodes. Microfluidic Devices and Systems III.

[33] Wang X.-B., Huang Y., Becker F.F., Gascoyne P.R.C. (1994). A unified theory of dielectrophoresis and travelling wave dielectrophoresis. J. Phys. D Appl. Phys..

[34] Lei U., Lo Y.J. (2011). Review of the theory of generalised dielectrophoresis. IET Nanobiotechnol..

[35] Sarkar P., Nicholson P.S. (1996). Electrophoretic deposition (EPD): Mechanisms, kinetics, and application to ceramics. J. Am. Ceram. Soc..

[36] Besra L., Liu M. (2007). A review on fundamentals and applications of electrophoretic deposition (EPD). Prog. Mater. Sci..

[37] Van der Biest O.O., Vandeperre L.J. (1999). Electrophoretic deposition of materials. Annu. Rev. Mater. Sci..

[38] Neirinck B., Van der Biest O., Vleugels J. (2013). A current opinion on electrophoretic deposition in pulsed and alternating fields. J. Phys. Chem. B.

[39] Markx G.H., Huang Y., Zhou X.F., Pethig R. (1994). Dielectrophoretic characterization and separation of micro-organisms. Microbiology.

[40] Fan S.-K., Huang P.-W., Wang T.-T., Peng Y.-H. (2008). Cross-scale electric manipulations of cells and droplets by frequency-modulated dielectrophoresis and electrowetting. Lab Chip.

[41] Braschler T., Demierre N., Nascimento E., Silva T., Oliva A.G., Renaud P. (2008). Continuous separation of cells by balanced dielectrophoretic forces at multiple frequencies. Lab Chip.

[42] Kirby B.J. (2010). Micro-and Nanoscale Fluid Mechanics: Transport in Microfluidic Devices.

[43] Brady J.F. (2011). Particle motion driven by solute gradients with application to autonomous motion: Continuum and colloidal perspectives. J. Fluid Mech..

[44] Feynman R.P., Leighton R.B., Sands M. (2011). The Feynman Lectures on Physics, Vol. I: The New Millennium Edition: Mainly Mechanics, Radiation, and Heat.

[45] Landau L.D., Bell J.S., Kearsley M.J., Pitaevskii L.P., Lifshitz E.M., Sykes J.B. (2013). Electrodynamics of Continuous Media.

[46] Sun J. (2012). Pulse-width modulation. Dynamics and Control of Switched Electronic Systems.

[47] COMSOL Multiphysics^®^ v. 5.3.

[48] Cicuta P., Donald A.M. (2007). Microrheology: A review of the method and applications. Soft Matter.

[49] Gal N., Lechtman-Goldstein D., Weihs D. (2013). Particle tracking in living cells: A review of the mean square displacement method and beyond. Rheol. Acta.

[50] Dopico A. (2007). Methods in Membrane Lipids.

[51] Pethig R. (2010). Dielectrophoresis: Status of the theory, technology, and applications. Biomicrofluidics.

[52] Jones T.B., Jones T.B. (2005). Electromechanics of Particles.

[53] (2017). Programming Reference Manual, COMSOL Multiphysics^®^ v. 5.3.

[54] (2017). COMSOL AC/DC Module User’s Guide, COMSOL Multiphysics^®^ v. 5.3.

[55] (2017). COMSOL Particle Tracing Module User’s Guide, COMSOL Multiphysics^®^ v. 5.3.

[56] (2017). COMSOL Multiphysics Reference Manual, COMSOL Multiphysics^®^ v. 5.3.

[57] Einstein A. Investigations on the Theory of the Brownian Movement. http://www.citeulike.org/group/8865/article/4036262.

[58] Jones T.B., Washizu M. (1994). Equilibria and dynamics of DEP-levitated particles: Multipolar theory. J. Electrost..

[59] Washizu M., Jones T.B. (1994). Multipolar dielectrophoretic force calculation. J. Electrost..

[60] (2016). MATLAB.

[61] Hibbeler R.C. (2001). Engineering Mechanics.

